# NF1 with 47,XYY mosaicism diagnosed by mandibular neurofibromas

**DOI:** 10.1038/s41439-024-00279-8

**Published:** 2024-05-16

**Authors:** Erina Tonouchi, Kei-ichi Morita, Yosuke Harazono, Kyoko Hoshino, Tetsuya Yoda

**Affiliations:** 1 Department of Maxillofacial Surgery, Graduate School of Medical and Dental Sciences, Tokyo Medical and Dental University, Tokyo, Japan; 2 Bioresource Research Center, Tokyo Medical and Dental University, Tokyo, Japan; 3 Segawa Memorial Neurological Clinic for Children, Tokyo, Japan

**Keywords:** Disease genetics, Medical genetics

## Abstract

Neurofibromatosis type 1 (NF1) is an autosomal dominant nevus disease characterized by multiple manifestations, primarily café-au-lait macules and neurofibromas. Here, we present the case of an NF1 patient with 47,XYY mosaicism whose diagnosis was prompted by café-au-lait macules on the skin and mandibular neurofibromas. Targeted next-generation sequencing of the patient’s blood sample revealed a novel frameshift mutation in *NF1* (NM_000267.3:c.6832dupA:p.Thr2278Asnfs*8) that is considered a pathogenic variant.

Neurofibromatosis type 1 (NF1; OMIM 162200), an autosomal dominant nevus disease with multiple manifestations, mainly café-au-lait macules and neurofibromas, affects 1 in 3000 people, and approximately 50% of cases arise de novo^1^. The causative gene, *NF1* (17q11.2; NM_001042492.3), was identified in 1990^2–4^ and encodes the GTPase-activating protein (GAP) neurofibromin^5^. The diagnostic criteria for NF1 were outlined at the National Institutes of Health (NIH) Consensus Development Conference in 1988^6^ and revised in 2021^7^. Approximately 7% of NF1-related neurofibromas manifest in the oral cavity, and mandibular occurrence is rare^8^. The incidence of 47,XYY syndrome in live-born male infants is 1 in 1000^9^, and the disorder is commonly diagnosed in the first decade of life based on developmental delays, behavioral issues, and tall stature^10^. Herein, we report a novel *NF1* frameshift mutation in a patient with NF1 and 47,XXY/46,XY mosaicism whose NF1 diagnosis was prompted by mandibular neurofibromas.

At 7 years of age, the patient visited Tokyo Medical and Dental University Hospital’s oral surgery department following a radiolucent finding around a buried left mandibular first molar on radiographic examination. His medical history included epilepsy, intellectual disability, autism spectrum disorder, and a mosaic karyotype of 47,XYY (43.3%)/46,XY (56.7%), identified through chromosome karyotyping at age 1. The patient’s parents showed no abnormalities, and the family history was unremarkable. The left mandibular first molar had not erupted, and panoramic radiography revealed a radiolucent area around its crown (Fig. 1a). A biopsy confirmed the presence of neurofibroma around the left mandibular first molar. Skin examination revealed multiple café-au-lait macules throughout the body. Clinical diagnosis of NF1 was based on these macules and multiple neurofibromas observed in the left mandibular coronoid process, left mandibular ramus, left mandibular body (biopsy area), and adipose tissue of the left masticator space on computed tomography (CT) scans at age 9 (Fig. 1b–e). Jaw neurofibromas were managed through observation. However, given that the left mandibular first molar had not erupted, the tumor in that area was removed, and fenestration surgery was performed at 10 years of age. Magnetic resonance imaging (MRI) at 16 years of age revealed a right pontine hamartomatous lesion associated with NF1 (Fig. 2a), a 35 mm neurofibroma in the medial mandibular ramus (Fig. 2b), and suspected neurofibromas in the right buccal (Fig. 2c) and left mandibular subcutaneous (Fig. 2b) regions. MRI at age 22 showed lumbar subcutaneous adenomas (Fig. 2d) and neurofibromas around the bilateral psoas major muscle (Fig. 2e) and along the pleura (Fig. 2f). By age 29, axillary and inguinal freckling was confirmed, prompting genetic testing of a peripheral blood sample. This study was approved by the Ethics Review Committee of our institution (D2020-084), and written informed consent was obtained from the parents of the patient. Targeted next-generation sequencing of *NF1*, *NF2*, *SPRED1*, *SMARCB1*, and *LZTR1* using the hybrid capture method at the Kazusa DNA Research Institute, Chiba, Japan, identified a heterozygous mutation, c.6832dupA (p.Thr2278Asnfs*8), in the *NF1* gene (NM_000267.3). Sanger sequencing confirmed that the patient had the same mutation (Supplementary Information 1). This *NF1* frameshift variant was not registered in ClinVar (https://www.ncbi.nlm.nih.gov/clinvar/), gnomAD (https://gnomad.broadinstitute.org/), ToMMo (https://jmorp.megabank.tohoku.ac.jp/), or HGMD (https://www.hgmd.cf.ac.uk/ac/index.php). The mutant was a null variant, absent from the gnomAD database, and assumed to be de novo, corresponding to PVS1, PM2, and PM6 in the American College of Medical Genetics and Genomics criteria, respectively; therefore, this variant was considered pathogenic^11^. With no notable changes in intramandibular or perimandibular lesions or systemic subcutaneous lesions, the patient remained under observation.

**Fig. 1:**
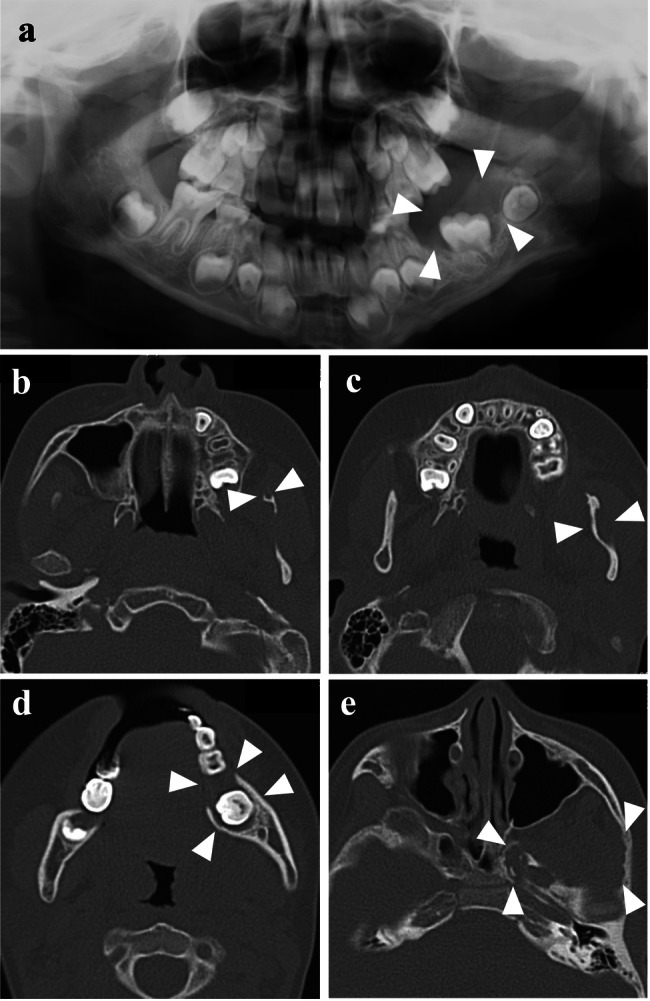
Panoramic radiography and CT imaging of the mandible. **a** Panoramic radiography at the initial examination (age 7). **b–e** CT imaging revealed multiple neurofibromas at age 9 in the left mandibular coronoid process (**b**), left mandibular ramus (**c**), left mandibular body (**d**), and adipose tissue of the left masticator space (**e**). Arrowheads indicate neurofibromas.

**Fig. 2:**
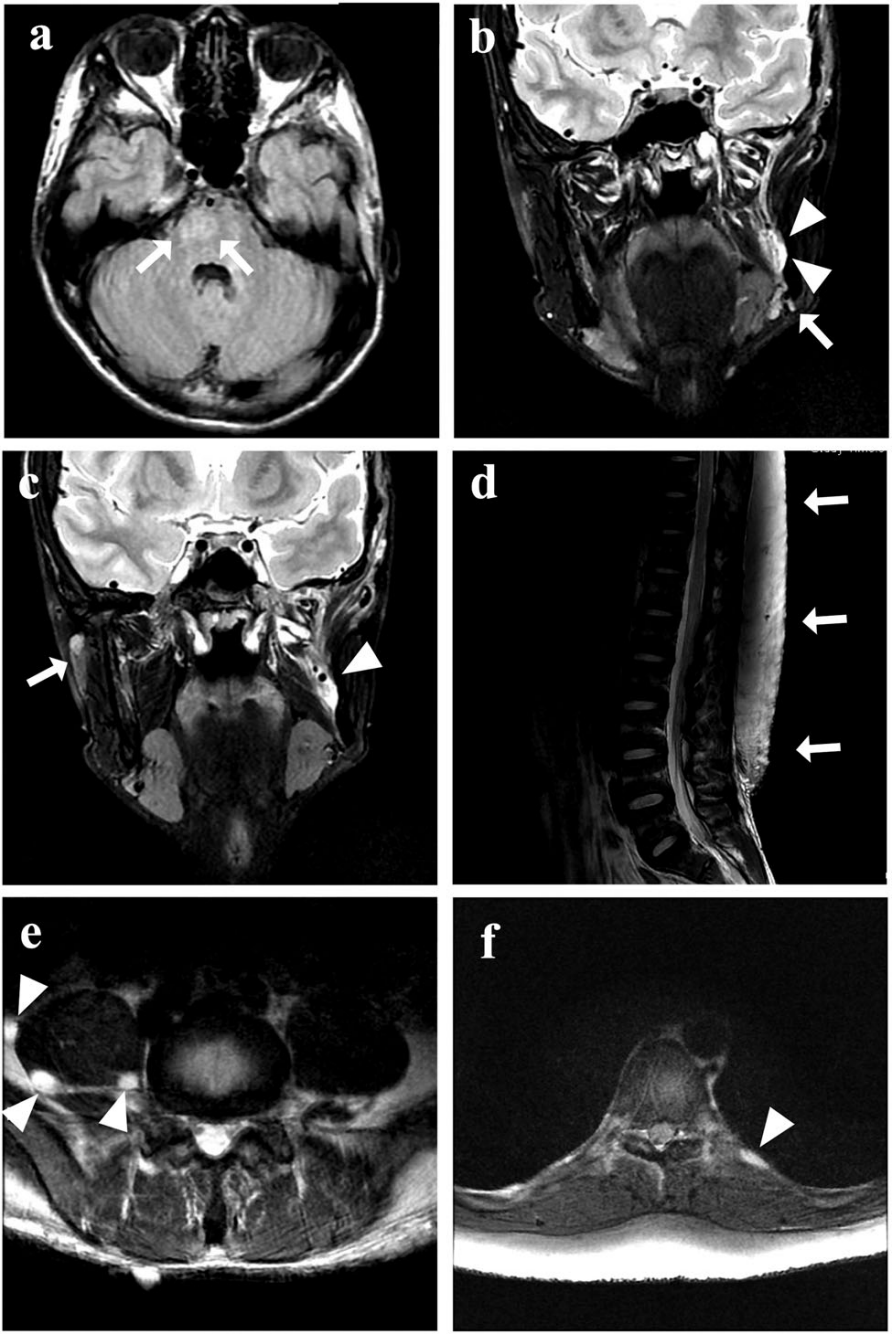
MRI findings. **a** Brain MRI axial FLAIR image at age 16. Arrows indicate hamartomatous lesions. **b**, **c** Coronal T2-weighted brain MR image at age 16. The arrows indicate nodules suspected to be neurofibromas in the left mandibular (**b**) and right buccal (**c**) subcutaneous areas. Arrowheads indicate neurofibromas. **d** Sagittal T2-weighted view of a lumbar MR image at age 22. The arrows indicate lumbar subcutaneous adenomas. **e**, **f** Axial T2-weighted views of a lumbar MR image at age 22. The arrows indicate neurofibromas around the bilateral psoas major muscle (**e**) and along the pleura (**f**).

Neurofibromatosis induces various abnormalities, particularly neurofibromas in the skin and nerves. In this patient, the NF1 diagnosis arose based on the presence of mandibular neurofibromas and café-au-lait macules on the skin. ~7% of neurofibromas in patients with NF1 are reported in the oral cavity^8^, with the palate, gingiva, tongue, buccal mucosa, and lips being common sites; however, mandibular occurrence is rare^8^. In the present case, despite the presence of café-au-lait macules as an NF1 clinical manifestation, the diagnosis was established only when mandibular neurofibromas were identified. Other developmental delays were noted, potentially attributed to the 47,XYY mosaic. Early recognition of comorbidities and comprehensive clinical assessments, facilitated by a timely diagnosis via a multidisciplinary approach, contribute to improving long-term health prognoses.

The diagnosis of 47,XYY is often based on behavioral and learning disabilities^3^. Despite its prevalence (1 in 1000 boys), ~85% of cases are undiagnosed until infertility arises due to atypical symptoms^9^, and limited information is available regarding the phenotype^10^. In our patient, chromosome karyotyping was performed at age 1 owing to developmental delays; however, the 43.3% mosaic nature of 47,XYY complicates our understanding of its impact on the patient’s clinical manifestations.

NF1, the first human condition attributed to pathogenic variants in RAS pathway genes, falls within the broader group of RASopathies, which includes Noonan, Costello, and cardiofaciocutaneous syndromes^12^. Legius syndrome (OMIM 611431), caused by a heterozygous pathogenic variant of *SPRED1*, is a RASopathy that exhibits the most overlap with NF1^13^. Because other syndromes exhibit clinical manifestations similar to those of NF1, a simultaneous search for candidate pathogenic genes, including *SPRED1*, is useful for confirming genetic diagnoses. Although our case involved a relatively easy clinical diagnosis of NF1, simultaneous gene searches are crucial in patients with insufficient clinical manifestations for disease differentiation.

The variable phenotype of NF1, even in patients with identical genetic variants, poses challenges in predicting disease severity, including within families^1,14^. This variability may be attributed to factors such as genetic modifiers, epigenetic abnormalities, and environmental influences^14^. Clinical genetic testing, which has high detection rates and utility in identifying genotype–phenotype correlations^5^, has become crucial. In the present case, the identified frameshift mutation, with a stop codon behind the RAS-GAP domain, was revealed through next-generation sequencing.

Previous studies have aimed to identify genotype–phenotype correlations that can be used to predict disease progression. However, mutation hotspots are lacking, and knowledge of genotypes associated with specific phenotypes is limited^15^. Scala et al. reported that missense mutations were inversely associated with neurofibromas, whereas frameshift mutations and whole-gene deletions were associated with skeletal abnormalities^16^. Gjorgjievska et al. reported positive correlations between cognitive impairment and gross deletions or truncating variants^17^. Napolitano et al. reported increased odds ratios for learning disabilities in patients carrying frameshift mutations^15^.

Symptom appearance varies, with café-au-lait macules and neurofibromas potentially manifesting by age 1, whereas other symptoms may develop in childhood, requiring up to 20 years for a patient to meet all diagnostic criteria. Recent advancements in NF1 genetic testing have provided a clinically available approach with a high detection rate, enabling earlier diagnosis in suspected patients.

## HGV database

The relevant data from this Data Report are hosted at the Human Genome Variation Database at 10.6084/m9.figshare.hgv.3400.

## Supplementary information

Supplementary information 1

## References

[1] Dunning-Davies, B. M. & Parker, A. P. Annual review of children with neurofibromatosis type 1. *Arch. Dis. Child. Educ. Pract. Ed.* **101**, 102–111 (2016). 26486853 10.1136/archdischild-2014-308084

[2] Viskochil, D. et al. Deletions and a translocation interrupt a cloned gene at the neurofibromatosis type 1 locus. *Cell* **62**, 187–192 (1990). 1694727 10.1016/0092-8674(90)90252-a

[3] Cawthon, R. M. et al. A major segment of the neurofibromatosis type 1 gene: cDNA sequence, genomic structure, and point mutations. *Cell* **62**, 193–201 (1990). 2114220 10.1016/0092-8674(90)90253-b

[4] Wallace, M. R. et al. Type 1 neurofibromatosis gene: identification of a large transcript disrupted in three NF1 patients. *Science* **249**, 181–186 (1990). 2134734 10.1126/science.2134734

[5] Koczkowska, M. et al. Clinical spectrum of individuals with pathogenic NF1 missense variants affecting p.Met1149, p.Arg1276, and p.Lys1423: genotype–phenotype study in neurofibromatosis type 1. *Hum. Mutat.* **41**, 299–315 (2020). 31595648 10.1002/humu.23929 PMC6973139

[6] Neurofibromatosis. Conference statement. National Institutes of Health Consensus Development Conference. *Arch. Neurol.* **45**, 575–578 (1988). 3128965

[7] Legius, E. et al. Revised diagnostic criteria for neurofibromatosis type 1 and Legius syndrome: an international consensus recommendation. *Genet. Med.* **23**, 1506–1513 (2021). 34012067 10.1038/s41436-021-01170-5 PMC8354850

[8] Kunisada, Y., Yoshioka, N., Ibaragi, S., Okui, T., Nagatsuka, H. & Sasaki, A. A case of intramandibular neurofibroma resembling a radicular cyst in a neurofibromatosis type 1 patient. *Int. J. Surg. Case Rep.* **82**, 105883 (2021). 33878668 10.1016/j.ijscr.2021.105883 PMC8081929

[9] Zou, C., Yu, D., Geng, H., Lan, X. & Sun, W. A patient with 47, XYY mosaic karyotype and congenital absence of bilateral vas deferens: a case report and literature review. *BMC Urol.* **22**, 16 (2022). 35109852 10.1186/s12894-022-00965-1 PMC8809031

[10] Bardsley, M. Z. et al. 47,XYY syndrome: clinical phenotype and timing of ascertainment. *J. Pediatr.* **163**, 1085–1094 (2013). 23810129 10.1016/j.jpeds.2013.05.037 PMC4097881

[11] Richards, S. et al. Standards and guidelines for the interpretation of sequence variants: a joint consensus recommendation of the American College of Medical Genetics and Genomics and the Association for Molecular Pathology. *Genet. Med.* **17**, 405–424 (2015). 25741868 10.1038/gim.2015.30 PMC4544753

[12] Rauen, K. A. The RASopathies. *Annu. Rev. Genom. Hum. Genet.* **14**, 355–369 (2013). 10.1146/annurev-genom-091212-153523 PMC4115674 23875798

[13] Brems, H. et al. Germline loss-of-function mutations in SPRED1 cause a neurofibromatosis 1-like phenotype. *Nat. Genet.* **39**, 1120–1126 (2007). 17704776 10.1038/ng2113

[14] Szudek, J., Joe, H. & Friedman, J. M. Analysis of intrafamilial phenotypic variation in neurofibromatosis 1 (NF1). *Genet. Epidemiol.* **23**, 150–164 (2002). 12214308 10.1002/gepi.1129

[15] Napolitano, F. et al. Genotype–phenotype correlations in neurofibromatosis type 1: identification of novel and recurrent NF1 gene variants and correlations with neurocognitive phenotype. *Genes (Basel)* **13**, 1130 (2022). 35885913 10.3390/genes13071130 PMC9316015

[16] Scala, M. et al. Genotype–phenotype correlations in neurofibromatosis Type 1: a Single-Center Cohort Study. *Cancers (Basel)* **13**, 1879 (2021). 33919865 10.3390/cancers13081879 PMC8070780

[17] Gjorgjievska, M., Bozhinovski, G., Sukarova-Angelovska, E., Kocova, M., Kanzoska, L. M. & Plaseska-Karanfilska, D. Mutational spectrum and genotype–phenotype correlations in neurofibromatosis type 1 patients from north Macedonia: identification of ten novel NF1 pathogenic variants. *Balk. Med. J.* **40**, 252–261 (2023). 10.4274/balkanmedj.galenos.2023.2022-12-28 PMC10339848 37073110

