# Supplementary material for: NF1 with 47,XYY mosaicism diagnosed by mandibular neurofibromas
Source: Hum Genome Var. 2024 May 16;11:22. doi: 10.1038/s41439-024-00279-8 (PMC11099053; doi:10.1038/s41439-024-00279-8)

Supplementary Information 1

NF1 mutation. A Sanger sequencing electropherogram from the patient demonstrates the same mutation identified by targeted next-generation sequencing, c.6832dupA. Primer sequences for *NF1* mutation are as follows: forward primer 5′-TTCCTGAATTCATTCCGAGATT-3′; reverse primer 5′- TTCAACACTGATACCCAAAATGA -3′.

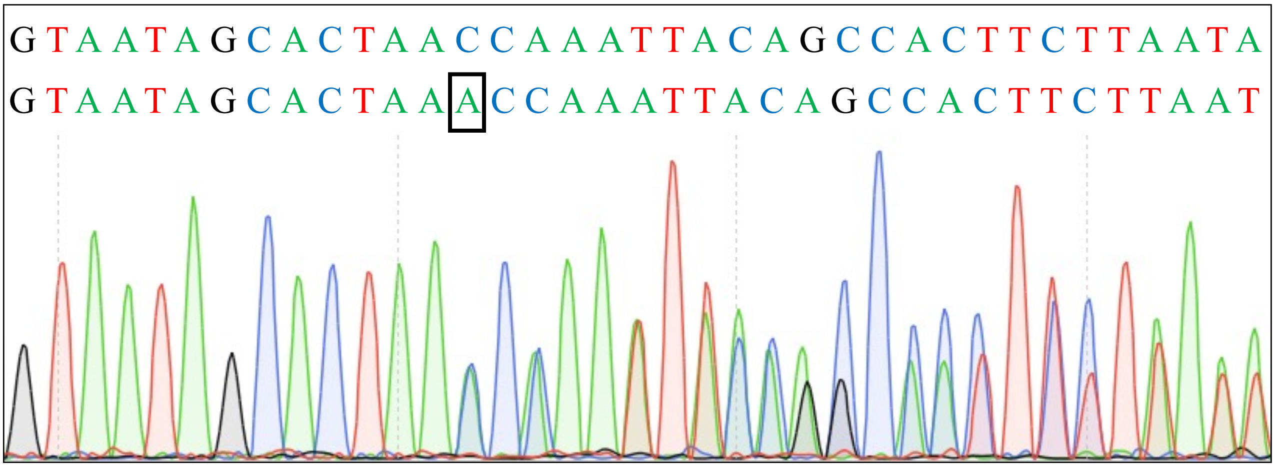

Supplement: Supplementary file 1 — Supplementary information 1 [file 41439_2024_279_MOESM1_ESM.pdf]
